# Pattern, barriers, and predictors of mental health care utilization among Egyptian undergraduates: a cross-sectional multi-centre study

**DOI:** 10.1186/s12888-023-04624-z

**Published:** 2023-03-06

**Authors:** Mohamed Baklola, Mohamed Terra, Mohamed A. Elzayat, Doaa Abdelhady, Abdel-Hady El-Gilany, ARO team of collaborators

**Affiliations:** 1 Public Health and Community Medicine Department, Faculty of Medicine, Mansoura University, 60El-Gomhoria Street, Mansoura, 35516 Egypt; 2 Faculty of Medicine, Mansoura University, 60El-Gomhoria Street, Mansoura, 35516 Egypt; 3 Alpha Research Organization (ARO), Mansoura, Egypt

**Keywords:** Mental health, University students, Egypt, Psychological distress, Help-seeking, Barriers

## Abstract

**Background:**

Mental health disorders are a major public health concern especially among undergraduates, globally and within Egypt. Most individuals suffering from mental illnesses either do not seek care at all or seek it only after a large delay. It is therefore critical to identify the barriers that prevent them from seeking professional help to solve the problem from its roots. Thus, the objectives of the study were to assess the prevalence of psychological distress, the need for professional mental health care, and the barriers to seeking available services among undergraduate students in Egypt.

**Methods:**

A proportionate allocation technique was used to recruit 3240 undergraduates from 21 universities. Symptoms of psychological distress were evaluated using the Arabic General Health Questionnaire (AGHQ-28) and a score of above nine was used to identify positive cases. The pattern for utilization of mental health care was assessed using a multi-choice question and barriers to mental health care were assessed using the Barriers to Access to Care Evaluation (BACE- 30) tool. Logistic regression was adopted to identify the predictors of psychological distress and seeking professional health care.

**Results:**

The prevalence of psychological distress was 64.7% and the need for professional mental health care was 90.3% of those with psychological distress. The top barrier to receiving professional mental health services was wanting to solve the problem on their own. Logistic regression revealed that female sex, living away from family and positive family history of mental disorders were independent predictors of psychological distress. Students from urban backgrounds were more likely to seek help than students from rural ones. While age above 20 and positive family history of mental disorders were independent predictors for seeking professional help. There is no significant difference between medical and non-medical students in terms of psychological distress.

**Conclusion:**

The findings of the study showed there is a high prevalence of psychological distress and a lot of instrumental and attitudinal related barriers to seeking mental health care and highlighted the urgent need to develop interventions and preventive strategies to address the mental health of university students.

## Background

The prevalence of mental health problems is rising worldwide, and in recent decades, there has been a progressive rise in focus on the mental health and well-being of university students [1].

The transition from high school to university is a critical developmental period for undergraduate students of constant psychosocial and academic changes; during this period anxious and depressive symptoms as well as the exacerbation of previous mental health disorders are [2, 3]. The high prevalence of mental health disorders among university students is identified as a challenge under the third Sustainable Development Goal (SDG), which seeks to promote health and well-being to all people at all stages of their [4]. A previous study, conducted in Ethiopia, showed that almost one-third of undergraduates were experiencing mental [5].

Most mental disorders begin in early adulthood and are linked to a long delay in treatment [6]. In Africa, only 14% of people who suffer from mental illnesses get treated [7]. Indicative of the widespread stigma surrounding mental health treatment, this is a clear example of its pervasiveness. Untreated or insufficiently treated mental illness is linked to the development of more serious conditions, dropping out of school, becoming addicted, and harming oneself [8]. The transition to university from high school corresponds with a high-risk period for maladaptive coping, the onset of psychopathology and academic failure consequently. However, it also provides an essential window of opportunity for prompt prevention and intervention [9].

In Egypt, primary care services are delivered through two tiers, the Family Health Unit (FHU) and the Family Health Center (FHC). The FHU consists of healthcare professionals such as doctors, nurses, social workers, and health educators, while the FHC also includes specialist physicians and a core healthcare team. Mental health services are managed by the General Secretariat of Mental Health and Addiction Treatment (GSMHAT), a governmental body that oversees 18 mental health hospitals and provides training to service providers [10]. In 2017, there were 5391 primary care units, with 9022 physicians assigned, though the target was 16,000. Mental health services are provided through multiple systems, including the GSMHT, mental health departments in general hospitals, psychiatric departments in public university medical schools, and military hospitals [10]. The GSMHAT currently manages 18 hospitals and centers in 13 governorates, with 5237 beds and 22 outpatient clinics [10]. However, access to mental health care in Egypt faces several challenges, including a shortage of healthcare providers, inadequate coordination between governmental departments, and a high prevalence of mental health issues and addiction [11]. Improving access to mental health care requires addressing these systemic issues, such as increasing the number of healthcare providers and enhancing inter-departmental collaboration [12].

The majority of research conducted at Egyptian universities is mainly concerned with determining the incidence of mental and psychological distress as opposed to examining potential barriers to seeking professional help. Furthermore, the literature is scarce about the assessment of the perceived need for mental health services and demographic characteristics related to barriers to receiving mental health care among Egyptian university students. Even though it is common knowledge that getting professional care is the first step toward a better life, many people still are unable to receive it because of financial, cultural, and other sources of limitations. For this reason, it is crucial to investigate and understand the various factors that prevent students from seeking help.

Therefore, the objectives of this study were to assess the prevalence of psychological distress, and the utilization of professional mental health care, and to identify barriers and demographic predictors to seeking available professional mental health services among undergraduate students in Egypt.

## Methods

### Study design and study period

A descriptive, cross-sectional study with an analytic component was conducted in the academic year 2021–2022 at 21 Egyptian universities.

### Sample size

The sample size was calculated using Medcalc 15.8. The primary outcome of interest is the percentage of students with psychological distress. A previous study conducted among undergraduates in Ethiopia found that the prevalence of mental distress was 34.6% [5]. Based on this study, with an alpha error of 5%, study power of 80%, and 5% precision with design effect 9, the calculated sample size was 3132 students.

### Sampling and data collection approach

A convenience sample was taken after determining the required sample size at each of the selected universities using the proportionate allocation technique. From January 1, 2022, until the sample size was reached, students completed the questionnaire using Google forms. The questionnaire was sent to all students from all academic years through official channels on the Telegram app and other social media platforms. They could also answer on their own time and anonymously.

### Measures

The survey questionnaire included four sections: Demographic Characteristics Questionnaire was used to record data such as participants’ sex, age, marital status, residence, living away from family, family history of mental illness, and parents’ level of education. The “Arabic General Health Questionnaire” (AGHQ-28), a structured self-reported online Arabic questionnaire, was used to screen for ICD-10 psychiatric disorders and to highlight the need for mental health treatment among students [13]. The AGHQ-28 is a 28-item measure of common mental health problems, including somatic symptoms (items 1–7), anxiety/insomnia (items 8–14), social dysfunctions (items 15–21), and severe depression (items 22–28). Based on the binary scoring method, a cut-off point of 9 was used to define “cases” and “non-cases” for presenting symptoms of psychiatric morbidity. The Arabic AGHQ has been shown to be a valid screening instrument in the Arab community with an overall sensitivity and specificity of 0.83 [13].

A question was used to assess the utilization of mental health services for those who screened positive in AGHQ as they need further confirmation by professionals. The question is phrased as follows: ‘What is the source of treatment do you use?’ with the response options of None, Professional, Religious, or Others.

In the data analysis phase, the data from students who had scored 9 or above on the AGHQ and did not utilize professional mental health care services was analyzed to identify the barriers that prevented them from accessing professional mental health care. The barriers to seeking professional care were assessed using the 30-item scale “Barriers to Access to Care Evaluation (BACE-III) Arabic Version” which has been shown to have acceptable levels of reliability and validity [14]. The BACE-III consists of stigma and attitudinal and instrumental barrier items. The scale rates barrier to mental health care on a Likert scale of 0 – Not at all, 1 – Somewhat, 2 – quite a lot, and 3 – a lot. The higher scores indicate a greater barrier. Five of the thirty items contain a fifth option: “Not applicable”. Findings for each barrier are presented in three ways: mean score for the item, no barrier at all, minor barrier (the percentage of answering 1 or 2), or major barrier (the percentage of answering 3) based on the BACE-III manual for researchers.

### Training of data collaborators

At each university, a team of collaborators was assembled. They were trained, by the authors through online meetings, uniformly on how to approach students online.

### Statistical analysis

Data were analysed using the Statistical Package for Social Science Program (SPSS 25 for Windows). To summarise the demographic characteristics of the participants and to identify barriers to mental health care services, descriptive statistical measures (i.e., percentage, frequency, mean, and standard deviation) were used. The Pearson chi-square test was used to examine the association between demographic variables and being positive for psychiatric illness and the need for professional care. In addition, binary logistic regression was used to model the relationship between demographic variables and participants who scored positively for psychiatric distress and who might require professional health care. The adjusted odds ratio was used to calculate the relative risk of each variable to the listed factors. Statistical significance was set at p-value of less than 0.05.

## Results

### Demographic characteristics and psychological distress

The questionnaire was completed by 3240 undergraduate students with females making up the vast majority (64.7%). Around two-thirds (68.1%) of the participants had a score of 9 or above on the AGHQ-28. The participants’ ages ranged from 17 to 26 years old, with a mean age of 20.68 years (SD = 1.93). In terms of marital status, 93.8% were single, with only 10.1% of the whole participants living alone away from their families. Moreover, more than half (58.2%) were from urban areas (Table 1). There is a statistically significant difference among individuals who screened positive for sex, a family history of mental illness, living alone, and treatment methods used.

**Table 1:** Demographic characteristics of the study participants and those with psychological distress

Variables	Total Sample (n) column % N (3240)	Screened positive for psychological distress (n) row % N (2207)	P value
Sex			**< 0.0001**
Male	1144 (35.3%)	696 (60.8%)	
Female	2096 (64.7%)	1511 (72.08%)	
Age			
Mean	20.68	20.58	
SD	1.925	1.89	
Minimum	17	17	
Maximum	26	26	
Residence			0.40
Urban	1886 (58.2%)	1296 (68.7%)	
Rural	1354 (41.8%)	911 (67.3%)	
Faculty			0.58
Medial	2334 (72%)	1583 (67.8%)	
Non-medical	906 (28%)	642 (70.9%)	
University			0.85
Nile Delta Universities	1714 (52.9%)	1165 (67.9%)	
Others	1526 (47.1%)	1042 (68.3%)	
Do you live alone			**0.02**
Yes	328 (10.1%)	242 (73.8%)	
No	2912 (89.9%)	1965 (67.5%)	
Marital Status			0.27
Single	3039 (93.8%)	2076 (68.3%)	
Ever married	201 (6.2%)	131 (65.2%)	
Father education level			0.48
Secondary or below	968 (29.9%)	668 (69%)	
Above secondary	2272 (70.1%)	1539 (67.7%)	
Mother education level			0.35
Secondary or below	1236 (38.1%)	854 (69.1%)	
Above secondary	2004 (61.9%)	1353 (67.5%)	
Methods of treatment used			**< 0.0001**
No	2373 (73.2%)	1575 (66.4%)	
Professional	313 (9.7%)	247 (78.9%)	
Religious	425 (13.1%)	289 (68%)	
Others	129 (4%)	96 (74.4%)	
Family history of mental disorder			**< 0.0001**
Yes	486 (15%)	381 (78.4%)	
No	2754 (85%)	1826 (66.3%)	

### Utilization of mental health care services

The prevalence of psychological distress was 68.1%. Female students had a higher rate (72.1%). Of the 2207 respondents, 90.3% didn’t use professional mental health care; 71.3% did not seek it at all, 13.1% sought religious methods, 11.2% sought professional mental health care, and 4.3% sought other methods. There is a statistically significant difference between those who didn’t use professional mental health care services based on their sex, area of residence, history of mental disorder, father’s educational level, and mother’s educational level (Table 2).

**Table 2:** The association between demographic variables and Seeking professional mental health care

Variables	Seeking professional mental health care			P value
	**Total** **(N = 2207)**	**Yes (n) row %** **(N = 247)**	**No (n) row %** **(N = 1960)**	
Sex				**0.02**
Male	696	62 (8.9%)	634 (91.1%)	
Female	1511	185 (12.2%)	1326 (87.8%)	
Residence				**< 0.0001**
Urban	1296	172 (13.3%)	1124 (86.7%)	
Rural	911	75 (8.2%)	836 (91.8%)	
Faculty				0.708
Medial	1583	180 (11.4%)	1403 (88.6%)	
Non-medical	624	67 (10.7%)	557 (89.3%)	
University				0.279
Nile Delta Universities	1165	122 (10.5%)	1043 (89.5%)	
Rest of Egypt	1042	125 (12%)	917 (88%)	
Do you live alone				0.829
Yes	242	28 (11.6%)	214 (88.4%)	
No	1965	219 (11.1%)	1746 (88.9%)	
Marital Status				0.146
Single	2076	225 (10.8%)	1851 (89.2%)	
Engaged	87	13 (14.9%)	47 (85.1%)	
Married	40	8 (20%)	32 (80%)	
Divorced	4	1 (25%)	3 (75%)	
Father education level				**0.001**
Secondary or below	668	53 (7.9%)	615 (92.1%)	
Above secondary	1539	194 (12.6%)	1345 (87.4%)	
Mother education level				**< 0.0001**
Low or no education	854	67 (7.8%)	787 (92.2%)	
High education	1353	180 (13.3%)	1173 (86.7%)	
Family history of mental disorder				**< 0.0001**
Yes	1826	92 (24.1%)	289 (75.9%)	
No	381	155 (8.5%)	1671 (91.5%)	

### Barriers to receiving professional mental health care for psychological distress

Of the 2207 participants who screened positive for psychological distress, 71.3% had not received any help and this might be due to the barriers to receiving mental health care as indicated in Table 3. We ordered the barriers based on the percentage of responses indicating the extent of the barrier (i.e., a little, quite a lot, and a lot). Subsequently, we selected the top five barriers as identified by the highest percentage.

**Table 3:** Barriers to receiving professional mental health care among students with psychological distress

Questions	Not a barrier (Not at all stopped me) (n) row %	Minor barrier (Stopped me a little) (n) row %	Major barrier (Stopped me quite a lot & a lot) (n) row %	Mean (SD)
Stigma-related barriers				
Concern that I might be seen as weak for having a mental health problem	788 (40.2%)	956 (48.8%)	216 (11.0%)	0.71 (0.654)
Concern that it might harm my chances when applying for jobs	810 (41.3%)	891 (45.5%)	259 (13.2%)	0.72 (0.683)
Concern about what my family might think	523 (26.7%)	952 (48.6%)	485 (24.7%)	0.98 (0.717)
Concern that I might be seen as ‘crazy’	1024 (52.2%)	707 (36.1%)	229 (11.7%)	0.59 (0.689)
Concern that I might be seen as a bad parent	993 (50.7%)	688 (35.1%)	279 (14.2%)	0.64 (0.719)
Concern that people I know might find out	784 (40.0%)	842 (43.0%)	334 (17.0%)	0.77 (0.72)
Concern that people might not take me seriously if they found out I was having professional care	763 (38.9%)	857 (43.7%)	340 (17.3%)	0.78 (0.719)
Not Wanting a mental health problem to be on my medical records	715 (36.5%)	859 (43.8%)	386 (19.7%)	0.83 (0.731)
Concern that my children may be taken into care or that I may lose access or custody without my agreement	1036 (52.9%)	585 (29.8%)	339 (17.3%)	0.64 (0.759)
Concern about what my friends might think, say, or do	739 (37.7%)	886 (45.2%)	335 (17.1%)	0.79 (0.711)
Concern about what people at work might think, say or do	718 (36.6%)	928 (47.3%)	314 (16.0%)	0.79 (0.696)
Feeling embarrassed or ashamed	972 (49.6%)	762 (38.9%)	226 (11.5%)	0.62 (0.683)
**Attitudinal-related barriers**				
**Dislike talking about my feelings, emotions, or thoughts**	**324 (16.5%)**	**941 (48.0%)**	**695 (35.5%)**	**1.19 (0.696)**
**Wanting to solve the problem on my own**	**212 (10.8%)**	**892 (45.5%)**	**856 (43.7%)**	**1.33 (0.661)**
**Concern about the treatments available (e.g., medication side effects)**	**428 (21.8%)**	**969 (49.4%)**	**563 (28.7%)**	**1.07 (0.708)**
Afraid of being put in hospital against my will	711 (36.3%)	724 (36.9%)	525 (26.8%)	0.91 (0.789)
Thinking that professional care probably would not help	758 (38.7%)	903 (46.1%)	299 (15.3%)	0.77 (0.696)
Thinking I did not have a problem	699 (35.7%)	942 (48.1%)	319 (16.3%)	0.81 (0.694)
Preferring to get help from family or friends	619 (31.6%)	936 (47.8%)	405 (20.7%)	0.89 (0.715)
Preferring to get alternative forms of care (e.g., traditional / religious healing or alternative /complementary therapies)	478 (24.4%)	1034 (52.8%)	448 (22.9%)	0.98 (0.687)
Having had previous bad experiences with professional care for mental health	1571 (80.2%)	313 (16.0%)	76 (3.9%)	0.24 (0.509)
**Instrumental-related barriers**				
Being too unwell to ask for help	1237 (63.1%)	603 (30.8%)	120 (6.1%)	0.43 (0.606)
Not being able to afford the financial costs involved	664 (33.9%)	838 (42.8%)	458 (23.4%)	0.89 (0.749)
Difficulty taking time off education	522 (26.6%)	951 (48.5%)	487 (24.8%)	0.98 (0.717)
Problems with transport or travelling to appointments	796 (40.6%)	873 (44.5%)	291 (14.8%)	0.74 (0.699)
**Being unsure where to go to get professional care**	**399 (20.4%)**	**923 (47.1%)**	**638 (32.6%)**	**1.12 (0.717)**
**Having no one who could help me get professional care**	**463 (23.6%)**	**932 (47.6%)**	**565 (28.8%)**	**1.05 (0.723)**
Professionals from my own ethnic or cultural group not being available	731 (37.3%)	931 (47.5%)	298 (15.2%)	0.78 (0.690)

* N = 1960

The most commonly cited barrier to seeking mental health care was “Wanting to solve the problem on my own” which was identified by 45.5% to some degree and 43.7% as a major barrier. The second was “Dislike talking about my feelings, emotions, or thoughts” which was identified by 48% to some degree and by 35.5% as a major barrier. The third barrier was “Being unsure where to go to get professional care”, which 47.1% of participants regarded as a difficulty to some degree and 42.6% regarded as a difficulty to a major degree. The fourth was “Concern about the treatments available”, which was identified as a barrier to some degree by 49.4% of participants, and 28.7% reported it as a major barrier. The fifth barrier was ‘Having no one who could help me get professional care’ reported as a barrier to any degree by 47.6%, while 48.8% of the participants identified it as a major barrier. Three of the top five barriers were attitudinal-related, while the third and fifth ones were instrumental-related (Table 3). Regarding stigma-related barriers, the most cited one was “Concern about what my family might think” which was identified by 48.6% to some degree and by 24.7% as a major barrier.

### Predictors of being positive in AGHQ-28 and seeking professional mental health care

Using Logistic Regression, the independent predictors for being positive in AGHQ-28, were positive family history of mental disorders, female sex, and living away from family with an adjusted odds ratio of 1.77, 1.66, and1.47 respectively. While the independent predictors for seeking professional help were positive family history of mental disorder and age above 20 with an adjusted odds ratio of 3.14 and 1.78; respectively (Table 4).

**Table 4:** Predictors of psychological distress and seeking professional help

	Positive for psychological distress (%)						
		**B**				**P-value**	**Adjusted OR (95%CI)**
Sex	Male			− 0.510		> 0.001	1 (r)
	Female						1.665 (1.427–1,942)
Living Away from Family	No			0.388		0.004	1 (r)
	Yes						1.474 (1.134–1.916)
Family history of mental disorder	Yes			0.575		> 0.001	1.776 (1.409–2.239)
	No						1 (r)
Constant					0.325	< 0.001	1.384
Note: Model X2 = 76.9 with P < 0.05, Percent corrected = 68.1%							
	**Seeking professional help (%)**						
			**B**			**P-value**	**Adjusted OR (95%CI)**
Sex	Male			0.310		0.052	1 (r)
	Female						1.363 (0.99–1.862)
Age	20 or below			0.579		< 0.001	1 (r)
	Above 20						1.784 (1.355–2.349)
Residence	Urban			0.257		0.101	1.293 (0.951–1.758)
	Rural						1 (r)
Father educational level	Secondary or below			0.185		0.374	1 (r)
	Above secondary						1.203 (0.800 -1.808)
Mother educational level	Low or no			0.355		0.070	1 (r)
	High						1.426 (0.971–2.095)
Family history of mental disorder	No			1.145		< 0.001	1 (r)
	Yes						3.144 (2.345–4.214)
Constant				-3.399		< 0.001	0.033

Note: Model X2 = 101.5 with P < 0.05, Percent corrected = 88.8%

## Discussion

The aim of this study was to examine the prevalence of psychological distress and seeking professional mental health care among undergraduate students in Egypt. Moreover, it aimed to identify demographic predictors and the barriers that interfere with the utilization of professional mental health services.

There is a high prevalence of psychological distress and possible psychological disorders among Egyptian university undergraduate students with 35.3% of them thinking about ending their own lives. The prevalence of psychological distress identified in this study (68.11%) is higher than that reported in a study conducted among Egyptian medical students at two different universities in Cairo, which showed that 63% of them had psychological distress [15]. Another study found that 38% of Menoufia University students experienced psychological distress [16]. The potential differences between these studies and our study could be attributed to the different cut-off points adopted, age groups, and settings.

In another study conducted in Saudi Arabia in 2018, 51.19% of undergraduate health professional students showed psychological distress [17]. In a study conducted in Kuwait in 2019, 50.47% of undergraduate students in health-related faculties exhibited psychological distress [18]. According to a study of university undergraduates in Ethiopia, 34.6% of students demonstrated psychological distress [5]. The difference between these studies and the current study could also be due to differences in study instruments used, a cut-off point used, cultures, and other sociodemographic details.

Another possible explanation for the higher rates in our study may be associated with the highly frequent barriers related to our study which made the problem worse over time. Another reason could be that most students who showed psychological distress did not receive ’mental health care help, which made the problem worse over time. This may not be surprising given that mental health services in Egypt are mostly delivered by hospitals, with minimal focus paid to integrate mental health into primary care [19]. This results in inadequate intervention, failure of early detection, and poor rehabilitation and social integration.

This study showed that females were considerably more likely than males to experience psychological distress, and so may require more concern in handling the problem. This is consistent with previous epidemiological studies that concluded that Egyptian females were more likely than males to experience psychological symptoms [20, 21]. One possible explanation for this sex difference may be that women in the MENA region are more prone to worry about the difficulties of their educational programs and to have fewer employment opportunities than males [21]. Also, living away from family may predict psychological distress. This may be due to the protective effect of a larger family network, as having more family members may provide more opportunities for social support and monitoring [22].

Having a family history of mental illness and being over the age of 20 were shown to be predictors for seeking professional mental health care in the logistic regression model. This association may be attributed to having a family history of mental disorders may increase their awareness of available mental health services and decrease the stigma associated with seeking help. This is consistent with prior research which showed that students with a family history of mental illness have a more positive attitude toward mental illness and are more likely to seek professional help [23].

Even though only a small number of students sought professional help, students from urban backgrounds were more likely to do so (13.3%) than students from rural ones (8.1%). A previous study found that psychiatric patients, particularly those from rural areas, frequently sought medical advice from traditional healers before or after receiving medical counsel from professionals [10]. Surprisingly, females (12.2%) were more likely to seek professional help than males (8.9%). This is consistent with a prior study showing that women had a higher intention to seek help [24]. Parents’ education was also associated with better mental health services seeking behaviors. This could be explained by having highly educated parents, who are mostly working as professionals with high salaries and are more able to obtain professional care. This results in their ability to encourage their children to seek costly mental health services, as well as a better understanding of mental health issues and how to manage them [25].

There was no significant difference between medical and non-medical students in terms of psychological distress. This contradicts a study that found medical students to be substantially more psychologically distressed than non-medical students [26]. A possible explanation may be those Egyptian students, particularly those who are not pursuing careers in the medical field, are constantly considering their futures, and are feeling increased pressure to find a decent career in order to be financially stable enough to marry and start their own families [27]. Moreover, there was no significant difference between medical and non-medical students regarding seeking professional mental health care services, despite the fact that they (medical students) should be more aware of the services provided. A previous study found that even though medical students have a responsibility to seek counselling for their health concerns, they frequently avoid disclosure and seek help for their concerns. [28]. This could be attributed to the social stigma attached to mental health even among medical students, who are perceived to be future doctors in their communities and the time constraints of these students.

The desire to handle problems on one’s own and having dislike of talking about one’s feelings were the first two of the top five stated barriers by students who did not seek any professional help. This may suggest that most students prefer to deal with their mental health issues on their own rather than seek professional help. This could be attributed to their perception of their illness as minor or temporary, doubt about the efficacy of professional mental health services, and fear of stigma [29]. As a result, people may prefer to manage their mental health problems on their own, maybe using coping strategies such as acceptance, religious methods, positive reframing, emotional support, denial, venting, self-blame, and substance use [30].

The third and fourth barriers to obtaining mental health services were a lack of knowledge about where to go to get professional care and concern about the treatments available respectively. This lack of knowledge may probably be caused by a lack of awareness campaigns of these services by the providers of these services. Moreover, mental health care in Egypt is offered by multiple systems, but it is still inadequate to support all the needs. Firstly, the Ministry of Health (MOH) manages only 18 hospitals and clinics in 14 of the total 29 governorates under the General Secretariat of Mental Health. Second, there are mental health departments in general hospitals that are administratively overseen by the MOH. In addition to a few private mental hospitals, non-governmental organisations (NGOs), and outpatient clinics around the whole country. Most of the public universities have a teaching referral hospital that provides mental health counselling and treatment to students as well as the general population with mental health issues [10]. Despite the increasing importance placed on mental health, access to mental health care in Egypt remains challenging. Some of the primary difficulties include a shortage of healthcare professionals, the lack of effective coordination between different government departments, and the high rates of mental health disorders and addiction [11]. Addressing these challenges is essential for improving access to mental health care. This can be achieved by increasing the number of mental health providers and improving collaboration between departments, thereby providing better care to those in need [12].

The fifth barrier was the lack of anyone who could help in obtaining professional care. This may also reflect a negative attitude of the general population about mental health issues and the lack of available knowledge at the university to support the students. Based on these five top barriers, we concluded that the most barrier to seeking mental health care services was the lack of knowledge, which is related to three of the top five barriers. As a result, we recommend close attention should be paid to this barrier.

Based on our findings, most of the barriers were instrumental and attitudinal related, while stigma was the lowest. Other studies depict that most of the barriers were attitudinal and stigma-related [31, 32]. However, this finding contrasts with the results of other studies [31, 33, 34], One study conducted among young adults aged 18–25 from the general UK population reported that stigma was the primary barrier to seeking mental health care, with 81% of participants stating that embarrassment and shame would prevent or delay them from seeking help [31]. This study emphasized the negative stigma attached to mental illness and how individuals were often judged and perceived differently once they disclosed their mental health issues. This is a good indicator that students are now less ashamed of having such conditions. This is in line with a cohort study on the predictors of help-seeking behaviour in people with mental health problems [24], which found that perceived stigma had no significant effect on help-seeking or assumptions about own help-seeking.

## Conclusion

Our study includes both strengths and weaknesses. This is the first study in Egypt to look at the barriers that prevent university students from obtaining professional mental health care. In order to make the results more generalizable and accurate, it included students from more than 21 universities and from various regions around the country. Furthermore, our study was not confined to a specific barrier, such as stigma; rather, we analysed the barriers from multiple perspectives, including stigma, attitude, and instrumental-related barriers. However, because the fact that the data in this study was self-reported may have introduced reporting bias and influenced the outcomes, some reporting bias may exist. Another disadvantage was that data was collected online, and this may have impacted the participants’ responses. So, in the future, it would be beneficial to use more objective methods of data collection to mitigate these limitations.

In conclusion, undergraduates in Egypt have a high prevalence of psychological distress and strong demand for professional mental health care services. Our findings may inform policymakers and stakeholders involved in psychiatric health and higher education issues. University students’ psychological well-being must be addressed with a focus on removing barriers that prevent them from seeking available services. Mental health care professionals in universities should raise awareness about mental health problems and services through information sessions and awareness campaigns, as well as highlight the benefits of receiving mental health care early and when and where to seek mental health services. Preventive strategies, in addition to interventions, are required to promote mental health. Offering more widespread and easily accessible mental health services for students may help. Universities also should screen their students for mental illness on a regular basis to identify students at high risk and support them as soon as possible. All of these factors may contribute to the development of active university-based mental health prevention and interventions that address the need for mental health services and overcome the above-mentioned key barriers.

## Data Availability

The datasets used during the current study are available from the corresponding author upon reasonable request.

## Declarations

### *ARO team of collaborators

Mohamed Baklola, Mohamed Terra, Mohamed A. Elzayat, Abdelmonem Siddiq, Norhan Ahmed, Rehab Baklola, Omnia Terra, Yousef Hawas, Salma Azab, Yara Eltaweel, Malek Talal, Mona Hamdy, Mohamed Osama, Mahmood Osman, Aya Ghallab, Mahmoud Baklola, Mai Mohamed, Mohamed Abdelaziz, Alaa Samy, Alshaimaa Ramadan Amer, Muhammad Elmanzlawey.
